# Supplementary material for: Real-time flow cytometry to assess qualitative and quantitative responses of oral pathobionts during exposure to antiseptics
Source: Microbiol Spectr. 2024 Aug 20;12(10):e00955-24. doi: 10.1128/spectrum.00955-24 (PMC11448261; doi:10.1128/spectrum.00955-24)
Supplement: Supplemental material — Fig. S1 to S7; Table S1. [file spectrum.00955-24-s0001.pdf]

Supplementary material:

Real-Time Flow Cytometry to assess qualitative and quantitative responses of oral pathobionts during exposure to antiseptics

Supplementary Figures

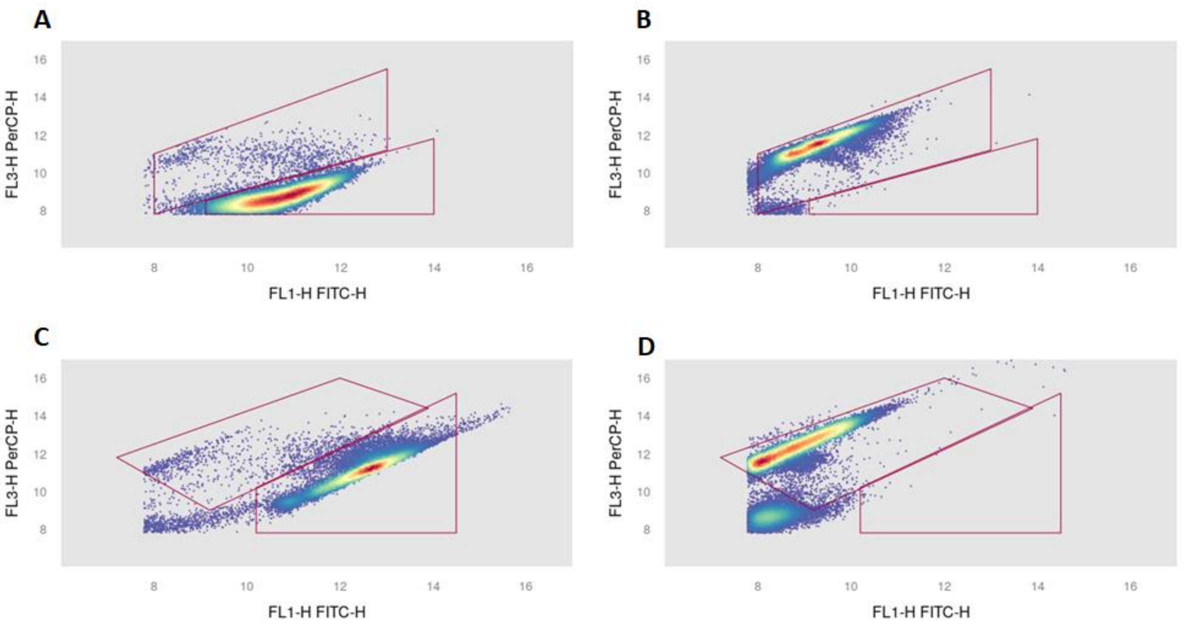

Figure S1: Examples of the manual gates that were drawn in the FL1/FL3 dot plots for the intact and damaged cell populations based on non-treated control (A, C) and heat-killed cells (B, D) for *A. actinomycetemcomitans* (A -B) and *S. mutans* (C- D)

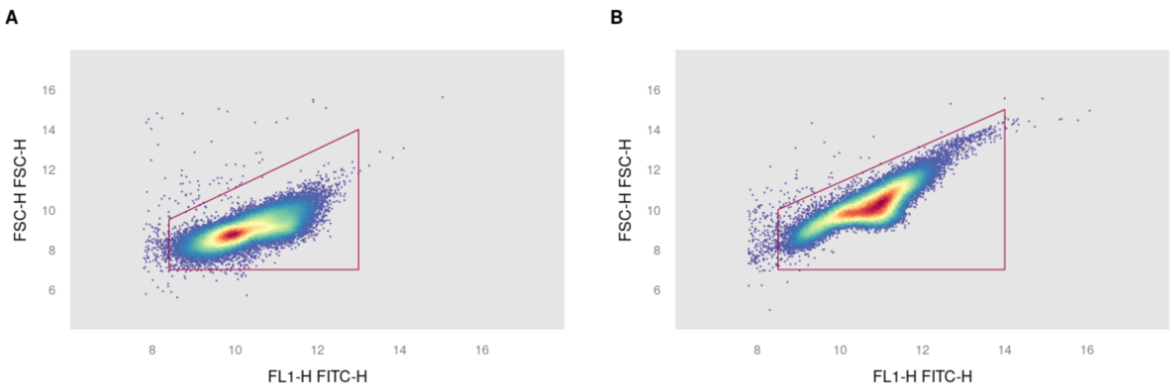

Figure S2: Examples of the manual gates that were drawn in the FL1/FSC dot plots for total cell populations to distinguish cells from background for *A. actinomycetemcomitans* (A) and *S. mutans* (B)

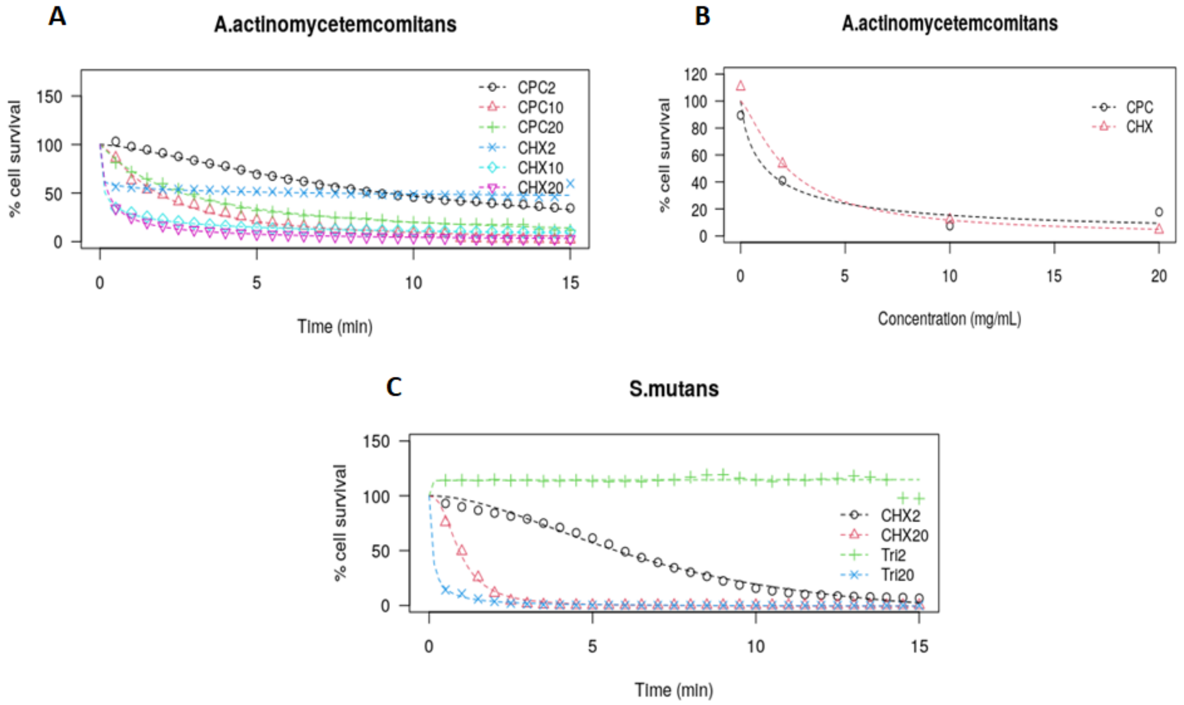

Figure S3: The log-logistic models fit on the percentage of surviving cells for a) *A. actinomycetemcomitans* over time for the different treatments (3 parameter log-logistic model) b) *A. actinomycetemcomitans* in different concentrations of the antiseptic at 10 min of treatment (3 parameter log-logistic model) and c) *S. mutans* over time for the different treatments (4 parameter log-logistic model)

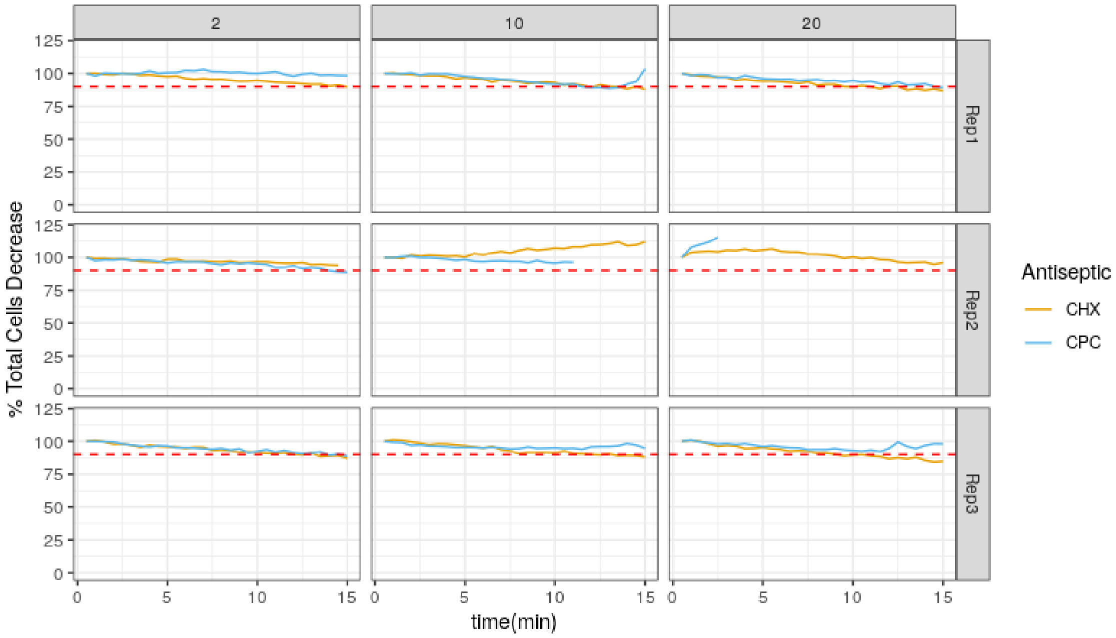

Figure S4: The percentage of total cells (intact and damaged cells) of *A. actinomycetemcomitans* over time compared to the first time point, for the three antiseptic concentrations and three replicates. The red dotted line represents 90% of the initial total cells.

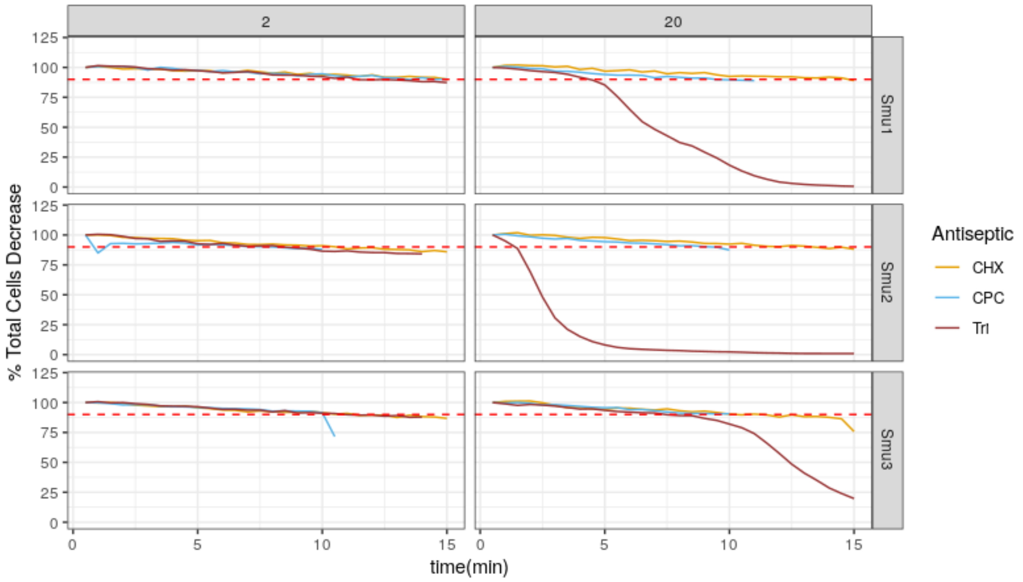

Figure S5: The percentage of total cells (intact and damaged cells) of *S. mutans* over time compared to the first time point, for the two antiseptic concentrations and three replicates. The red dotted line represents 90% of the initial total cells.

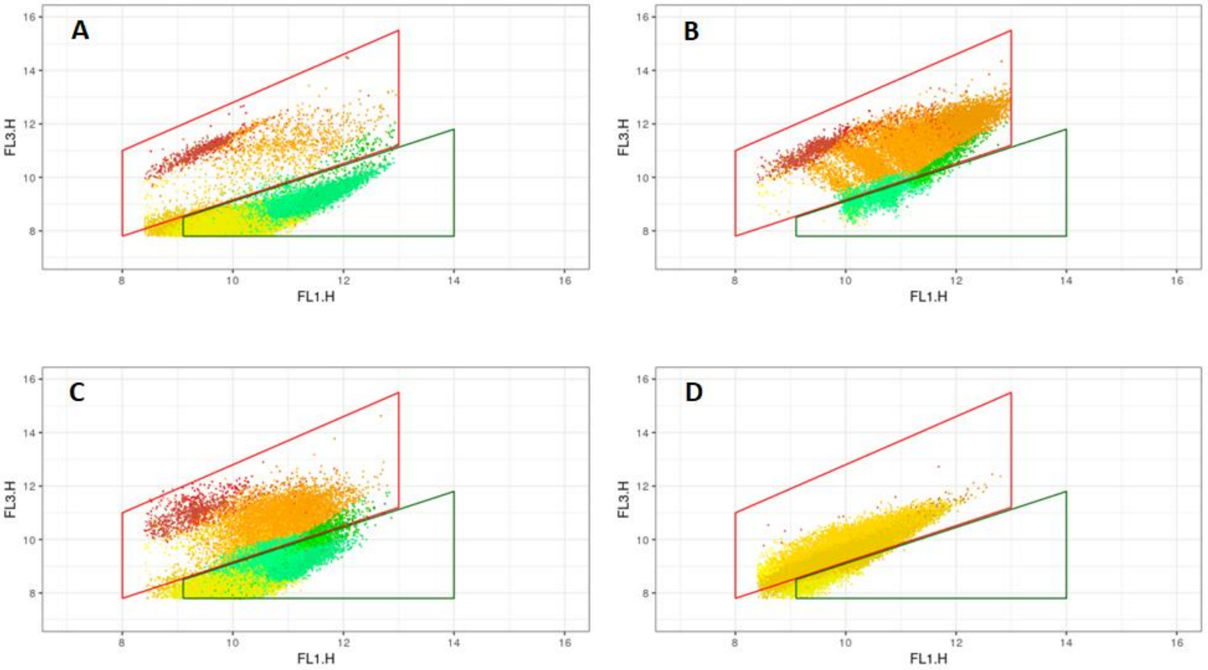

Figure S6: Examples of *A. actinomycetemcomitans* cells coloured according to the phenotypes as estimated based on the Gaussian Mixture model: a) untreated control after 4 min, B) cells treated with 20 mg/mL CHX after 4 min, C) cells treated with 2 mg/mL CPC after 4 min and D) cells treated with 20 mg/mL CPC after 4 min. The gates represent the manual gates that were drawn for the first part of the experiment: a) green for the intact cells and b) red for the damaged cells.

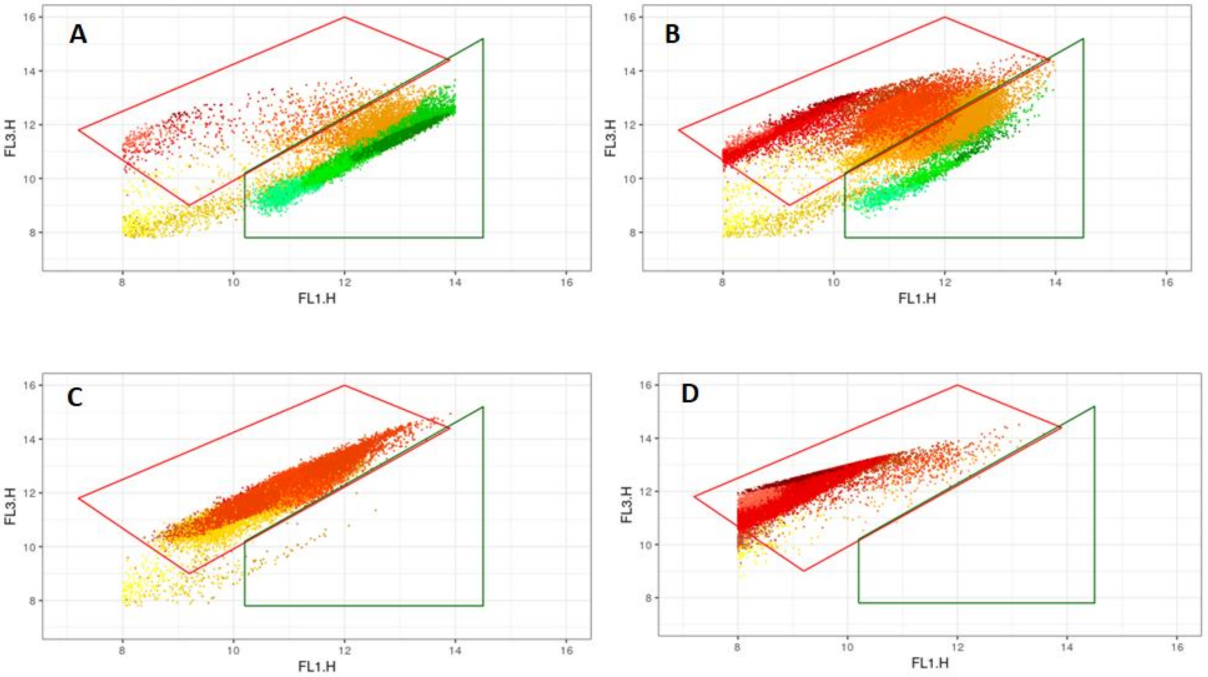

Figure S7: Examples of *S. mutans* cells coloured according to the phenotypes as estimated based on the Gaussian Mixture model: a) untreated control after 4 min, B) cells treated with 2 mg/mL CHX after 4 min, C) cells treated with 20 mg/mL CPC after 4 min and D) cells treated with 20 mg/mL triclosan after 4 min. The gates represent the manual gates that were drawn for the first part of the experiment: a) green for the intact cells and b) red for the damaged cells.

Supplementary Table 1: Minimum Inhibitory Concentrations (MIC) for CHX, CPC and triclosan for *A. actinomycetemcomitans* and *S. mutans*.

	CHX (µg/mL)	CPC (µg/mL)	Triclosan (µg/mL)
A. actinomycetemcomitans	1.25	5	0.3
S. mutans	0.15	0.6	10
